# Supplementary material for: Neutralization and Improvement of Bauxite Residue by Saline-Alkali Tolerant Bacteria
Source: Int J Environ Res Public Health. 2022 Sep 14;19(18):11590. doi: 10.3390/ijerph191811590 (PMC9517105; doi:10.3390/ijerph191811590)
Supplement: Supplementary file 1 [file ijerph-19-11590-s001.zip › ijerph-1884971-supplementary.pdf]

Supplementary Materials

Table S1. Change of the medium pH of different bacterial strains.

Bacterial strains	Genus	pH value after 48h	pH value after 72h
Blank		10.57	10.30
**ZH-1**	*Bacillus* sp.	**6.80**	**5.96**
ZH-2	*Bacillus* sp.	8.21	8.83
ZH-3	*Nesterenkonia* sp.	8.34	8.31
ZH-4	*Halomonas* sp.	7.82	7.79
ZH-5	*Bacillus* sp.	8.89	8.77
ZH-6	*Halomonas* sp.	8.32	8.27
ZH-7	*Nesterenkonia* sp.	8.84	8.82

Table S2. Factors and levels designed of $L_9(3^4)$ orthogonal experiment.

Level	Factors			
	A	B	C	D
1	2 g/L	2 g/L	0.3 g/L	0.3 g/L
2	5 g/L	3 g/L	0.5 g/L	0.5 g/L
3	8 g/L	4 g/L	0.7 g/L	0.7 g/L

Table S3. Analysis of the results using the range method for the pH decrease.

Case	A	B	C	D	pH
A1	2	2	0.3	0.3	8.40
A2	2	3	0.5	0.5	8.65
A3	2	4	0.7	0.7	8.98
A4	5	2	0.5	0.7	8.05
A5	5	3	0.7	0.3	5.97
A6	5	4	0.3	0.5	5.72
A7	8	2	0.7	0.5	6.03
A8	8	3	0.3	0.7	5.56
A9	8	4	0.5	0.3	5.58
*K*1	26.03	22.48	19.68	19.95	
*K*2	19.74	20.18	22.28	20.40	
*K*3	17.17	20.28	20.98	22.59	
*k*1（*K*1/3）	8.68	7.49	6.56	6.65	
*k*2（*K*2/3）	6.58	6.73	7.43	6.80	
*k*3（*K*3/3）	5.72	6.76	6.99	7.53	
R	2.96	0.76	0.87	0.88	
Order	A>D>C>B				
Optimal combination	$A_3B_2C_1D_1$				

Table S4. Correlation analysis between medium pH and organic acid concentration.

	Citric acid	Oxalic acid	Tartaric acid
pH(shake)	-0.885**	-0.878**	-0.733**
pH (static)	-0.932**	-0.663*	-0.845**

*Significance level of 0.05, **Significance level of 0.01.

Table S5. Correlation analysis between pH and MBC in bauxite.

residue.	MBC_{CK}	MBC_{RX}
pH_{CK}	-0.687	
pH_{RX}		-0.961**

**Significance level of 0.01.

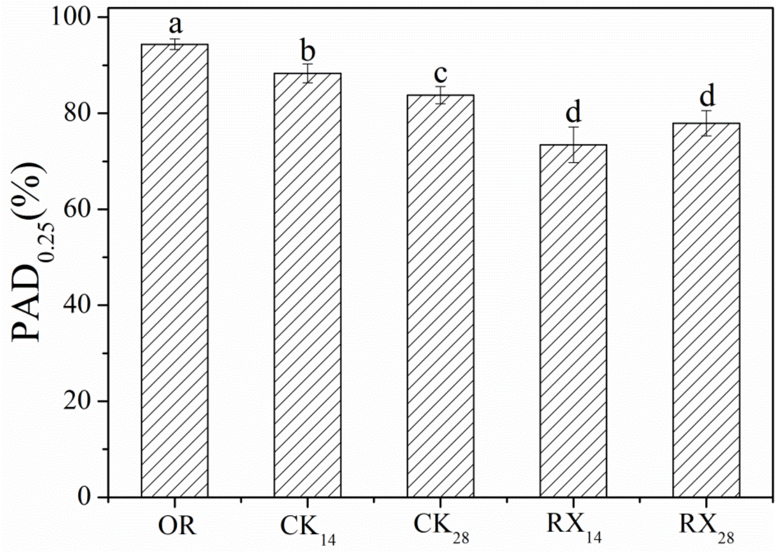

Figure S1. Aggregate stability of bauxite residue in different treatment. OR represent in the original bauxite residue; CK_{14} and CK_{28} represent the samples in treatment CK at 14 and 28 days, respectively; RX_{14} and RX_{28} represent the samples in treatment CK at 14 and 28 days, respectively.
